# Supplementary material for: Dynamic control of proinflammatory cytokines Il-1β and Tnf-α by macrophages in zebrafish spinal cord regeneration
Source: Nat Commun. 2018 Nov 7;9:4670. doi: 10.1038/s41467-018-07036-w (PMC6220182; doi:10.1038/s41467-018-07036-w)
Supplement: Supplementary file 1 — Supplementary Information [file 41467_2018_7036_MOESM1_ESM.pdf]

Tsarouchas et al.: Dynamic control of proinflammatory cytokines Il-1β and Tnf-α by macrophages in zebrafish spinal cord regeneration

Supplementary material: 3 Tables; 13 figures, 2 movie legends

Supplementary Table 1**:** List of mutations after CRISPR/Cas9 editing of *tnf-α.*

5’-GAAATTAGTAAACAGGGAGATTATCATTCCCGATG ATGGCATTTATTTTGTCTACAGCCAGGTGTCTTT-3’ **Wt**
5’-GAAATTAGTAAACAGGGAGATTATCATTCCCGAT - AATGGCATTTATTTTGTCTACAGCCAGGTGTCTTT-3’ **-1bp, +1bp**

5’-GAAATTAGTAAACAGGGAGATTATCATTCCCGATGATGGCATTTATTTTGTCTACAGCCAGGTGTCTTTG-3’ **Wt**
5’-GAAATTAGTAAACAGGGAGATTATCATTCCCG - - - ATGGCATTTATTTTGTCTACAGCCAGGTGTCTTTG-3’ **-3bp**

5’-GAAATTAGTAAACAGGGAGATTATCATTCCCGATG ATGGCATTTATTTTGTCTACAGCCAGGTGTCTTTG-3’ **Wt**
5’-GAAATTAGTAAACAGGGAGATTATCATTCCC - - - GATGGCATTTATTTTGTCTACAGCCAGGTGTCTTTG-3’ **-3bp**

5’-GAAATTAGTAAACAGGGAGATTATCATTCCCGATGATGGCAT TTATTTTGTCTACAGCCAGGTGTCT-3’ **Wt**
5’-GAAATTAGTAAACAGGGAGATTATCATTCCCGATG - - - - - - - GCATTATTTTGTCTACAGCCAGGTGTCT-3’ **-7bp, +3bp**

5’-GAAATTAGTAAACAGGGAGATTATCATTCCCGATG ATGGCATTTATTTTGTCTACAGCCAGGTG-3’ **Wt**
5’-GAAATTAGTAAACAGGGAGATTATCATTCCCGAT - AAATAAATGGCATTTATTTTGTCTACAGCCAGGTG-3’ **-1bp, +6bp**

5’-GAAATTAGTAAACAGGGAGATTATCATTCCCGATGATGGCATTTATTTTGTCTACAGCCAGGTGTCTTTG-3’ **Wt**
5’-GAAATTAGTAAACAGGGAGATTATC - - - - - - - - - - ATGGCATTTATTTTGTCTACAGCCAGGTGTCTTTG-3’ **-10bp**

5’-GAAATTAGTAAACAGGGAGATTATCATTCCCG ATGATGGCATTTATTTTGTCTACAGCCAGGTGT-3’ **Wt**
5’-GAAATTAGTAAACAGGGAGATTATCATTCCCGAAATTATGATGGCATTTATTTTGTCTACAGCCAGGTGT-3’ **+5bp**

5’-GAAATTAGTAAACAGGGAGATTATCATTCCCGATG ATGGCATTTATTTTGTCTACAGCCAGGTG-3’ **Wt**
5’-GAAATTAGTAAACAGGGAGATTATCATTCCCGATGGCATTTATGGCATTTATTTTGTCTACAGCCAGGTG-3’ **+6bp**

5’-GAAATTAGTAAACAGGGAGATTATCATTCCCGATG ATGGCATTTATTTTGTCTACAGCCAGGTGTCT-3’ **Wt**
5’-GAAATTAGTAAACAGGGAGATTATCATTCCCGATGGGAATGGCATTTATTTTGTCTACAGCCAGGTGTCT-3’ **+3bp**

5’-GAAATTAGTAAACAGGGAGATTATCATTCCCGATGAT GGCATTTATTTTGTCTA-3’ **Wt**
5’-GAAATTAGTAAACAGGGAGATTATCATTCCCGAT - - - AATGATAATCATTCCCGGCATTTATTTTGTCTA-3’ **-3bp, +16bp**

5’-GAAATTAGTAAACAGGGAGATTATCATTCCCGATGATGGCATTTATTTTGTCT ACAGCCAGGTGTCTTT-3’ **Wt**
5’-GAAATTAGTAAACAGGGAGATTATCATTCCCGAT - - - - - - - - - - - - - - - - - - - CACAGCCAGGTGTCTTT-3’ **-19bp, +1bp**

5’-GAAATTAGTAAACAGGGAGATTATCATTCCCGATGATG GCATTTATTTTGTCTACAGCCAGGTGTCTTT-3’ **Wt**
5’-GAAATTAGTAAACAGGGAGATTATCATTCCCGA - - - - - CTGCATTTATTTTGTCTACAGCCAGGTGTCTTT-3’ **-5bp, +2bp**

5’-GAAATTAGTAAACAGGGAGATTATCATTCCCGATG ATGGCATTTAT TTTGTCTACAGCC-3’ **Wt**
5’-GAAATTAGTAAACAGGGAGATTATCATTCCCGAT - - - - - - - - - - - - CTTCATTCCCATTTGTCTACAGCC-3’ **-12bp, +11bp**

Supplementary Table 2: List of primers used.

beta actin qPCR	Forward	5'-CACTGAGGCTCCCCTGAATCCC-3'
	Reverse	5'-CGTACAGAGAGAGCACAGCCTGG-3'
tgf-β1a qPCR	Forward	5'-GCTGTATGCGCAAGCTTTACA-3'
	Reverse	5'-GGACAATTGCTCCACCTTGTG-3'
il-1β qPCR	Forward	5'-ATGGCGAACGTCATCCAAGA-3'
	Reverse	5'-GAGACCCGCTGATCTCCTTG-3'
tgf-β3 qPCR	Forward	5'-AAAACGCCAGCAACCTGTTC-3'
	Reverse	5'-CCTCAACGTCCATCCCTCTG-3'
tnf-α qPCR	Forward	5'-TCACGCTCCATAAGACCCAG-3'
	Reverse	5'-GATGTGCAAAGACACCTGGC-3'
mpeg1 probe[1]	Forward	5′-GGATCCATCATGAAGTCAAG-3′′
	Reverse	5′-CTCGAGTACTTGAACCCGTG-3′′
il-1β gRNA	Forward	5'- ACTTCTGCTCAGCCTGTGTG-3'
	Reverse	5'- CCGCTGATCTCCTTGAGTACGAG-3'
tgf-β1a probe	Forward	5'-AAAGAGCCTGAATCCGGAGC-3'
	Reverse	5'-ACTTGCAGTTCCTCACCACC-3'
tnf-a gRNA	Forward	5'-ACCAGGCCTTTTCTTCAGGT-3'
	Reverse	5'-AGCGGATTGCACTGAAAAGT-3'
il-1β MO[2]	Forward	5'-TGCCGGTCTCCTTCCTGA-3'
	Reverse	5'-GCAGAGGAACTTAACCAGCT-3'
mmp2	Forward	5'-TGGCCGAAATGAACATGGTG-3'
	Reverse	5'-GGAACCCATCATCTCGACCC-3'
mmp9	Forward	5'-TACGGTAATGCTGAGGGTGC-3'
	Reverse	5'-GCCGTATCTCTGTTAGGGCA-3'
mmp11a	Forward	5'-CGCGAGATGCTTGCCTTTAC-3'
	Reverse	5'-AGCGCTGGATTTTGTAGGTGA-3'
mmp11b	Forward	5'-GCAGAGAGACGACCCACATT-3'

	Reverse	5’-ACCCAGTAATTCTGGCCTTGG-3’
mmp13a	Forward	5’-GCCAACAACCAGGTTTACAGTTAT-3’
	Reverse	5-TCTTCAGGCGGTAAGTATTAAAGAA-3’
mmp13b	Forward	5’-GCTCACGAGTTTGGTCATGC-3’
	Reverse	5’-CAGCCTCCAGTAAAACCTGTCT-3’
mmp14a	Forward	5’-TGAGTGAGGATGGAGCCAATG-3’
	Reverse	5’-GCGGTGACAGTAGAGTAGGC-3’
mmp14b	Forward	5’-AACGTCTGTATTCTCCCGCT-3’
	Reverse	5’-AGCCATGCCTCAGGTTTCAT-3’
mmp15a	Forward	5’-TTCAATGCGGAGTCTTGGCT-3’
	Reverse	5’-CTTTGGGCGAGTGGTTTTGG-3’
mmp15b	Forward	5’-AAAATCAGACTGGAGGTGGCA-3’
	Reverse	5’-TTGTTAGCAGATGACGGCGA-3’
mmp16a	Forward	5’-TGGACAACGAAACCAGCACT-3’
	Reverse	5’-AGAGCATAAACGCACCCCTC-3’
mmp16b	Forward	5’-GTGAGAGAACGGCAGGTAATG-3’
	Reverse	5’-GAATCCTCCGTCGCAGATGT-3’
mmp17a	Forward	5’-ATCTTTCCTGGAGGGTGAGGA-3’
	Reverse	5’-TGCAAACAGGTCCATCCCAT-3’
mmp17b	Forward	5’-CGGTCTGTAATGCGTCCGTA-3’
	Reverse	5’-ACATGCTCTGGCCTTTGAAGA-3’
mmp19	Forward	5’-TGTCGCACAACTAGGGTTTCA-3’
	Reverse	5’-AGCAGTGCCGAAGTGAATCT-3’
mmp20a	Forward	5’-CGCAACGAGTGGCATATGTT-3’
	Reverse	5’-TGAACGCGTCCATTGAGTTC-3’
mmp20b	Forward	5’-’ATTCCCATCAGGCGGACATC-3’
	Reverse	5’-GTCTCTTGGCCCGTAGAGC-3’
mmp23bb	Forward	5’-GCTCATGAAATCGGTCACGC-3’
	Reverse	5’-CTGCATCCAGAGGCTCGTAG-3’
mmp24	Forward	5’-CATGACGGCAATGACCTGTTC-3’

	Reverse	5’-GAGACTGACGCTCGTGTTTG-3’
mmp28	Forward	5’-TGCTATCAGAGAGTTTCAGTGGTT-3’
	Reverse	5’-CCTGACTGGTAGAAAGGCTCC-3’
mmp30	Forward	5’-GGTGTTCTGGCTCATGCAAA-3’
	Reverse	5’-TCGAACACCGTACAAAGCCT-3’

1 Ellett, F., Pase, L., Hayman, J. W., Andrianopoulos, A. & Lieschke, G. J. mpeg1 promoter transgenes direct macrophage-lineage expression in zebrafish. *Blood* **117**, e49 (2011).
2 Nguyen-Chi, M. *et al.* Transient infection of the zebrafish notochord with E. coli induces chronic inflammation. *Dis Model Mech* **7**, 871-882, doi:10.1242/dmm.014498 (2014).

Supplementary Table 3: List of antibodies used.

Antibody	Raised in	Source	Catalog #	Dilution
anti-GFP [1]	chicken	Abcam, Cambridge, USA	AB13970	1:300
Anti-4C4[2]	mouse	European Collection of Authenticated Cell Cultures (ECACC) , Salisbury UK	7.4.C4 (ECACC 92092321)	1:50
Monoclonal Anti-Tubulin, Acetylated[1]	mouse	Sigma	T6793	1:300
Anti-Tp63[1]	rabbit	Sigma	SAB2701838	1:300
Anti-Col I[1]	rabbit	Abcam	ab23730	1:300
Anti-HuC/HuD[3]	mouse	Invitrogen	A-21271	1:100
Anti-Mpx[4]	rabbit	GeneTex Irvine, California USA	GTX128379	1:300
Anti-Mfap4	rabbit	GeneTex	GTX132692	1:300
Anti-Il-1β [5]	rabbit	Proteintech, Manchester, UK	16806-1-AP	1:200
Anti-L-plastin[2]	rabbit	Yi Feng	-	1:500
Anti-TNFα[6]	rabbit	Anaspec	AS-55383	1:2000
Anti-α-Tubulin	mouse	DSHB, Iowa, USA		1:2.000
AffiniPure Fab Fragment Donkey Anti-Rabbit IgG (H+L)[7,8]	donkey	Jackson ImmunoResearch, Cambridge, UK	711-007-003	40 μg/ml
Cy5 Anti-rabbit	donkey	Jackson ImmunoResearch	711-175-152	1:300
Cy™3 Anti-Rabbit IgG (H+L)	donkey	Jackson ImmunoResearch	711-165-152	1:300
Cy™3 Aff Anti-Mouse IgG (H+L)	donkey	Jackson ImmunoResearch	715-165-150	1:300
Alexa Fluor® 488 Anti-Mouse IgG(H+L)	donkey	Jackson ImmunoResearch	715-545-150	1:300
Cy™5- Anti-Mouse IgG (H+L)	donkey	Jackson ImmunoResearch	715-175-150	1:300
Anti-rabbit	goat	LI-COR	IRdye680LT	1:10000
Anti-mouse	goat	LI-COR	IRdye800CW	1:10000

1 Wehner, D. *et al.* Wnt signaling controls pro-regenerative Collagen XII in functional spinal cord regeneration in zebrafish. *Nat Commun* **8**, 126, doi:10.1038/s41467-017-00143-0 (2017).

2 Ohnmacht, J. *et al.* Spinal motor neurons are regenerated after mechanical lesion and genetic ablation in larval zebrafish. *Development* **143**, 1464-1474, doi:10.1242/dev.129155 (2016).
3 Ulrich, F. *et al.* Reck enables cerebrovascular development by promoting canonical Wnt signaling. *Development* **143**, 147-159, doi:10.1242/dev.123059 (2016).
4 Kenyon, A. *et al.* Generation of a double binary transgenic zebrafish model to study myeloid gene regulation in response to oncogene activation in melanocytes. *Dis Model Mech* (2018).
5 Zheng, X. *et al.* Caffeine reduces hepatic lipid accumulation through regulation of lipogenesis and ER stress in zebrafish larvae. *J Biomed Sci* **22**, 105, doi:10.1186/s12929-015-0206-3 (2015).
6 Nelson, C. M. *et al.* Tumor necrosis factor-alpha is produced by dying retinal neurons and is required for Muller glia proliferation during zebrafish retinal regeneration. *J Neurosci* **33**, 6524-6539, doi:10.1523/jneurosci.3838-12.2013 (2013).
7 Laresgoiti, U. *et al.* Lung epithelial tip progenitors integrate glucocorticoid- and STAT3-mediated signals to control progeny fate. *Development* **143**, 3686-3699, doi:10.1242/dev.134023 (2016).
8 Prost, S., Kishen, R. E., Kluth, D. C. & Bellamy, C. O. Working with Commercially Available Quantum Dots for Immunofluorescence on Tissue Sections. *PLoS One* **11**, e0163856, doi:10.1371/journal.pone.0163856 (2016).

SUPPLEMENTARY FIGURES

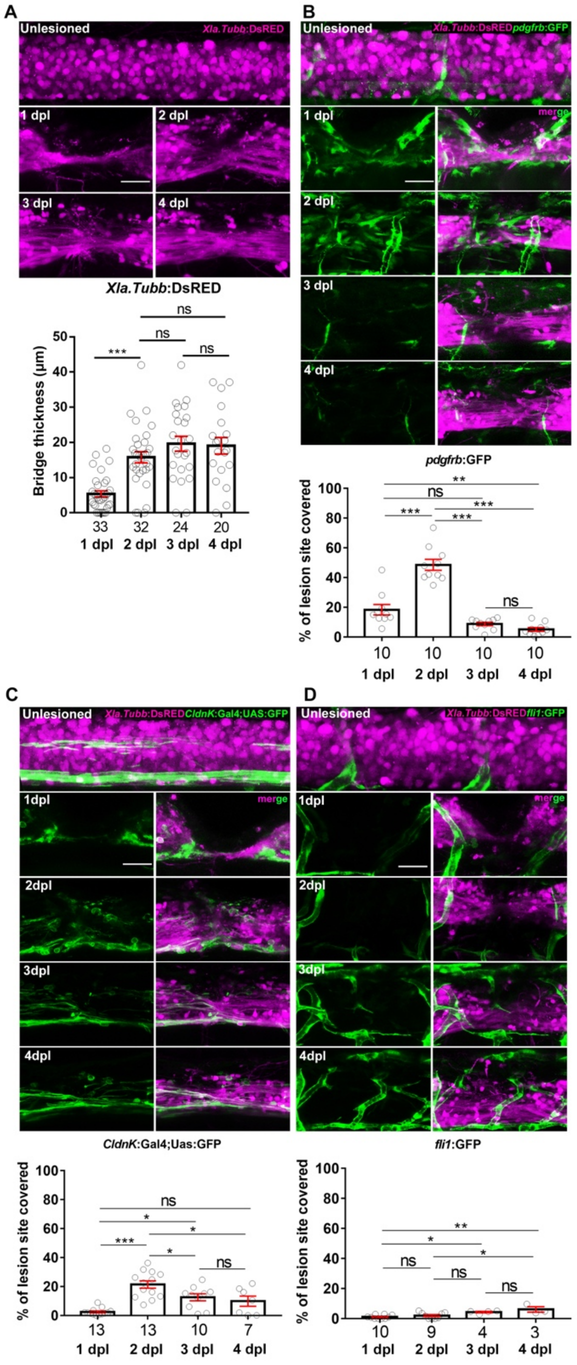

Supplementary Fig. **1**: Presence of fibroblast-like cells, but not myelinated cells or endothelial cells correlates with axonal regeneration. **A:** The thickness of the axonal bridge observed in *Xla.tubb*:DsRed transgenic animals increases after injury, starting at 1 dpl and plateauing at 2 dpl (*One-way ANOVA followed by Bonferroni multiple comparisons:* $F_{3,\ 107}$ = 17.77 ***P < 0.0001, ns indicates no significance). **B:** Presence of fibroblast-like cells (*pdfrb*:GFP+) in the lesion site is substantial from 1 dpl, it peaks at 2 dpl, and declines thereafter (*One-way ANOVA followed by Bonferroni multiple comparisons:* $F_{3,\ 36}$ = 53.92, **P<0.01,***P<0.0001, ns indicates no significance). **C:** Myelinating cells (*cldnK*:GFP+) appear in the injury site only at 2 dpl (*One-way ANOVA followed by Bonferroni multiple comparisons:* $F_{3,\ 39}$ = 14.52, *P0.05, ***P<0.0001, ns indicates no significance). **D:** Endothelial cells (*fli1*:GFP+) do not invade the lesion site (*One-way ANOVA followed by Bonferroni multiple comparisons*: $F_{3,\ 22}$ = 7.939, *P0.05, **P<0.01). Lateral views of the injury site are shown; rostral is left. Scale bar: 25 µm. Error bars indicate SEM.

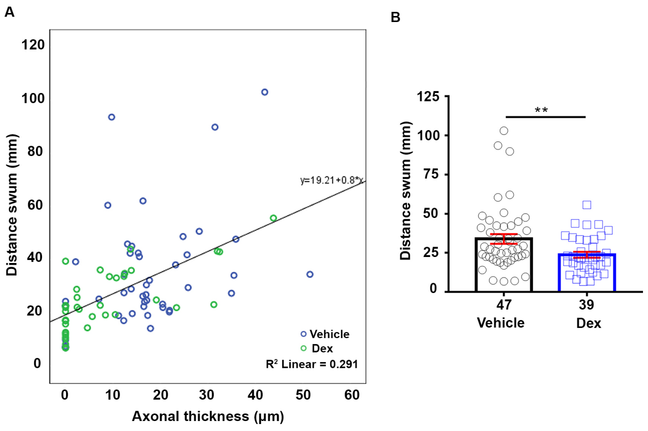

C

Correlations

		Distance swum (mm)	Axonal thickness (μm)	Treatment (Vehicle/Dex)
Distance swum (mm)	Pearson Correlation	1	.540**	-.284**
	Sig. (2-tailed)		.000	.008
	N	86	86	86
Axonal thickness (μm)	Pearson Correlation	.540**	1	-.367**
	Sig. (2-tailed)	.000		.001
	N	86	86	86
Treatment (Vehicle/Dex)	Pearson Correlation	-.284**	-.367**	1
	Sig. (2-tailed)	.008	.001	
	N	86	86	86

**. Correlation is significant at the 0.01 level (2-tailed).

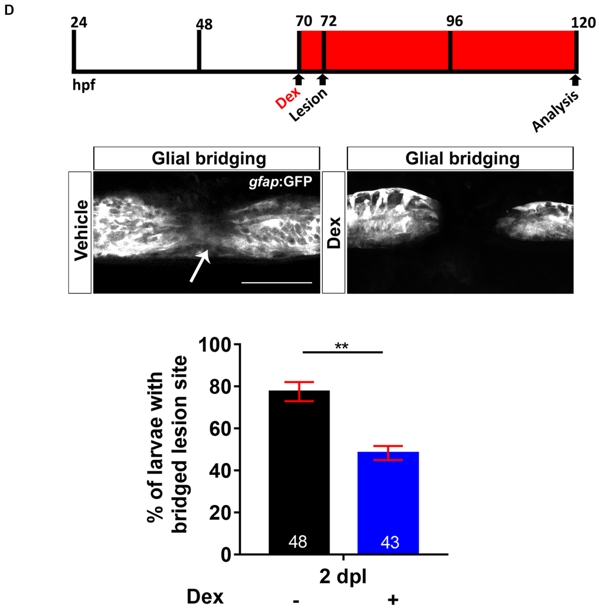

Supplementary Fig. **2**: Dexamethasone treatment impairs axonal and glial crossing of the injury site and recovery of touch-evoked swim distance. **A**: The thickness of the axonal bridge correlates with the distance swum after injury, following treatment with vehicle or dexamethasone. **B**: Distance swum is significantly impaired after treatment with dexamethasone (*t-test*:**P<0.01). **C**: Correlation table. There is a positive correlation between thickness of the axonal bridge and the distance swum, whereas there is a negative correlation between bridge thickness and dexamethasone treatment and between distance swum and dexamethasone treatment. **D**: Dexamethasone treatment impairs formation of glial bridges (arrow) over the injury site (Fisher's exact test: **P<0.01). Lateral views of the injury site are shown; rostral is left; Dex = dexamethasone. Scale bar in D: 100 μm. Error bars indicate SEM.

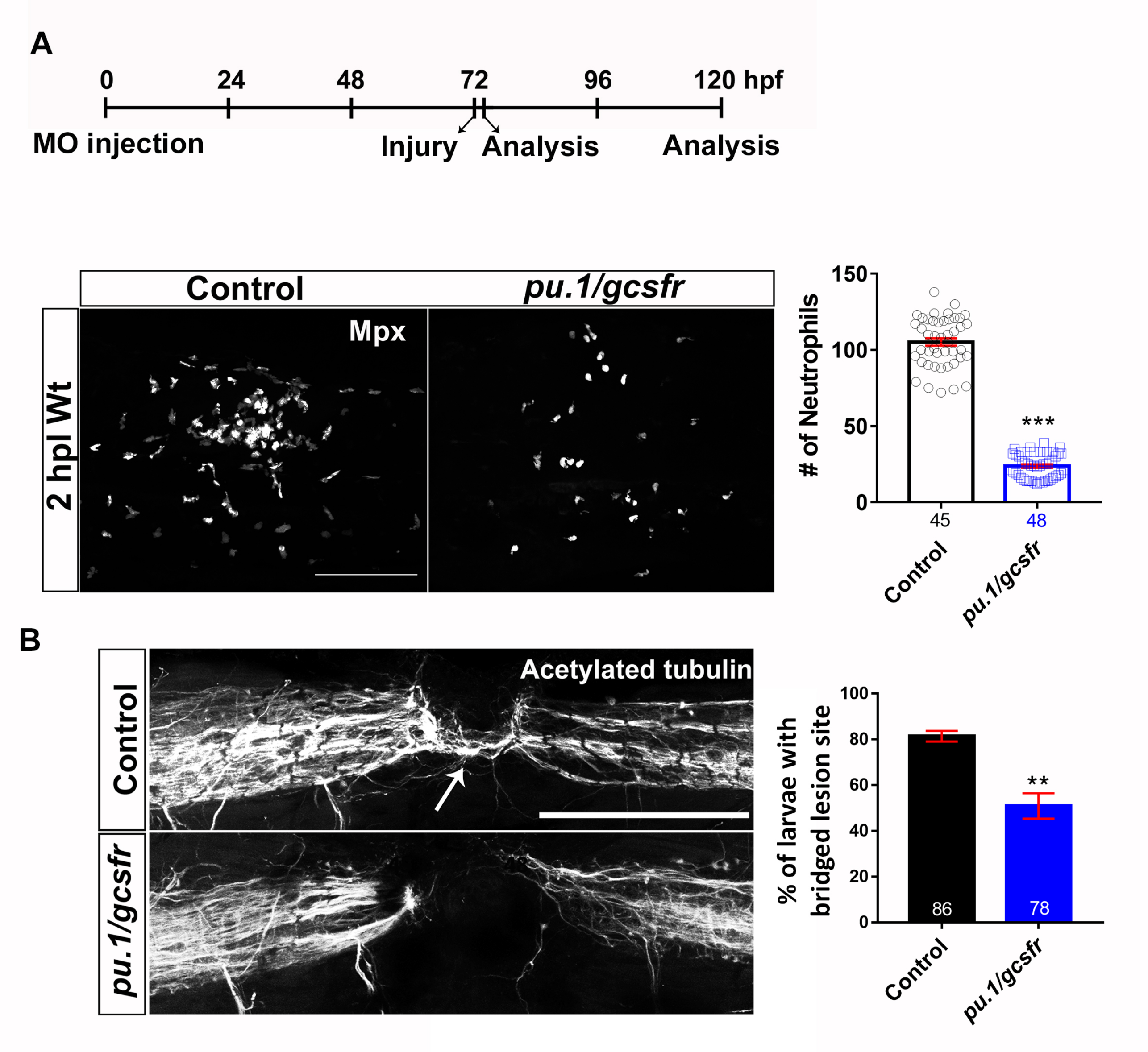

Supplementary Fig. **3**: Inhibiting immune cell development impairs axonal regeneration. **A:** Neutrophils are strongly reduced in number in *pu.1* and *gcsfr* double morpholino-injected animals (*Mann-Whitney test*: ***P<0.001). **B:** Quantification of axonal bridging (arrow; anti-acetylated Tubulin) shows that morpholino injection decreases the proportion of animals with axonal bridges (*Fisher's exact test*: **P<0.01). Lateral views of the injury site are shown; rostral is left. Scale bar: 100 µm. Error bars indicate SEM.

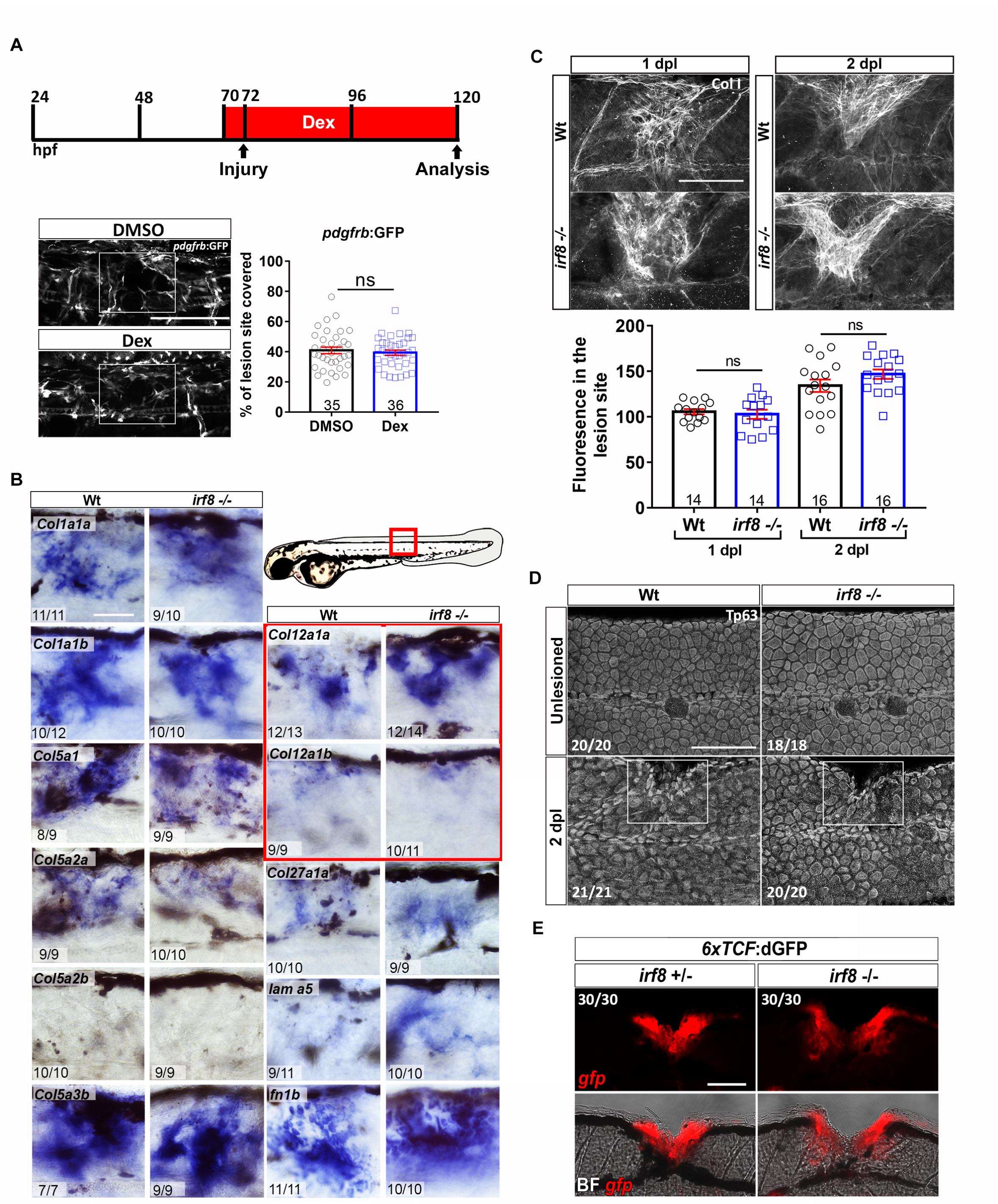

Supplementary Fig. **4**: Wnt-dependent ECM deposition and wound healing are not affected by compromising the immune reaction. **A:** The density of fibroblast-like cells (*pdgfrb*:GFP+) in the lesion site is not affected by inhibition of the immune response with dexamethasone (Dex) (*t-test*, ns = not significant). **B:** Expression of mRNAs for major ECM components, including *col12a1a* and *col12a1b* (red box) is not impaired in the absence of macrophages in *irf8* mutants. **C:** Immunoreactivity for Collagen I (Col I) is not altered in *irf8* mutants, compared to wildtype controls at 1 dpl and 2 dpl (*t-test*: ns indicated no significance). **D:** Basal keratinocytes (anti-Tp63+) cover the injury site (indicated by rectangle) in *irf8* mutants by 2 dpl, similar to wildtype animals. **E:** Activation of the Wnt pathway is unaltered in *irf8* mutants after injury. The *6xTCF*:dGFP reporter line crossed into the *irf8* mutants shows activation of Wnt signalling that is comparable to that in wildtype animals at 2 dpl. Lateral views of the injury site are shown.; rostral is left. Scale bars: 50 µm. Error bars indicate SEM.

A

Gene ID	Gene name	Wt *irf8-/-*
ZDB-GENE-070817-3	*mmp11a-matrix metallopeptidase 11a*	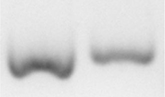
ZDB-GENE-070820-1	*mmp16a-matrix metallopeptidase 16a*	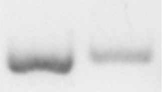
ZDB-GENE-061009-22	*mmp16b-matrix metallopeptidase 16b*	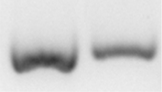
ZDB-GENE-040724-262	*mmp24-matrix metallopeptidase 24*	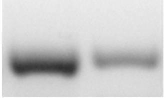
ZDB-GENE-100226-1	*mmp28-matrix metallopeptidase 28*	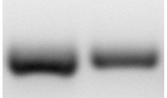
	β-actin	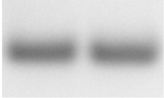

B

Not regulated

Gene ID	Gene name
ZDB-GENE-030131-9123	*mmp2-matrix metallopeptidase 2*
ZDB-GENE-040426-2132	*mmp9-matrix metallopeptidase 9*
ZDB-GENE-070817-2	*mmp11b-matrix metallopeptidase 11b*
ZDB-GENE-031202-2	*mmp13a-matrix metallopeptidase 13a*
ZDB-GENE-030131-6152	*mmp13b-matrix metallopeptidase 13b*
ZDB-GENE-030901-1	*mmp14a-matrix metallopeptidase 14a*
ZDB-GENE-030901-2	*mmp14b-matrix metallopeptidase 14b*
ZDB-GENE-070817-4	*mmp15a-matrix metallopeptidase 15a*
ZDB-GENE-070817-6	*mmp15b-matrix metallopeptidase 15b*
ZDB-GENE-070820-2	*mmp17a-matrix metallopeptidase 17a*
ZDB-GENE-081107-17	*mmp17b-matrix metallopeptidase 17b*
ZDB-GENE-100308-3	*mmp19-matrix metallopeptidase 19*
ZDB-GENE-100603-2	*mmp20a-matrix metallopeptidase 20a*
ZDB-GENE-100603-3	*mmp20b-matrix metallopeptidase 20b*
ZDB-GENE-050417-448	*mmp23bb-matrix metallopeptidase 23bb*
ZDB-GENE-060421-5765	*mmp30-matrix metallopeptidase 30*

Supplementary Fig. **5:** Some matrix metalloproteases showed reduced expression in *irf8* mutants. **A:** RT-PCR showed lower expression of 5 *mmp* genes in the injury site of *irf8* mutants compared to wildtype. **B:** *mmp* genes for which no robust change in *irf8* mutants could be detected are listed.

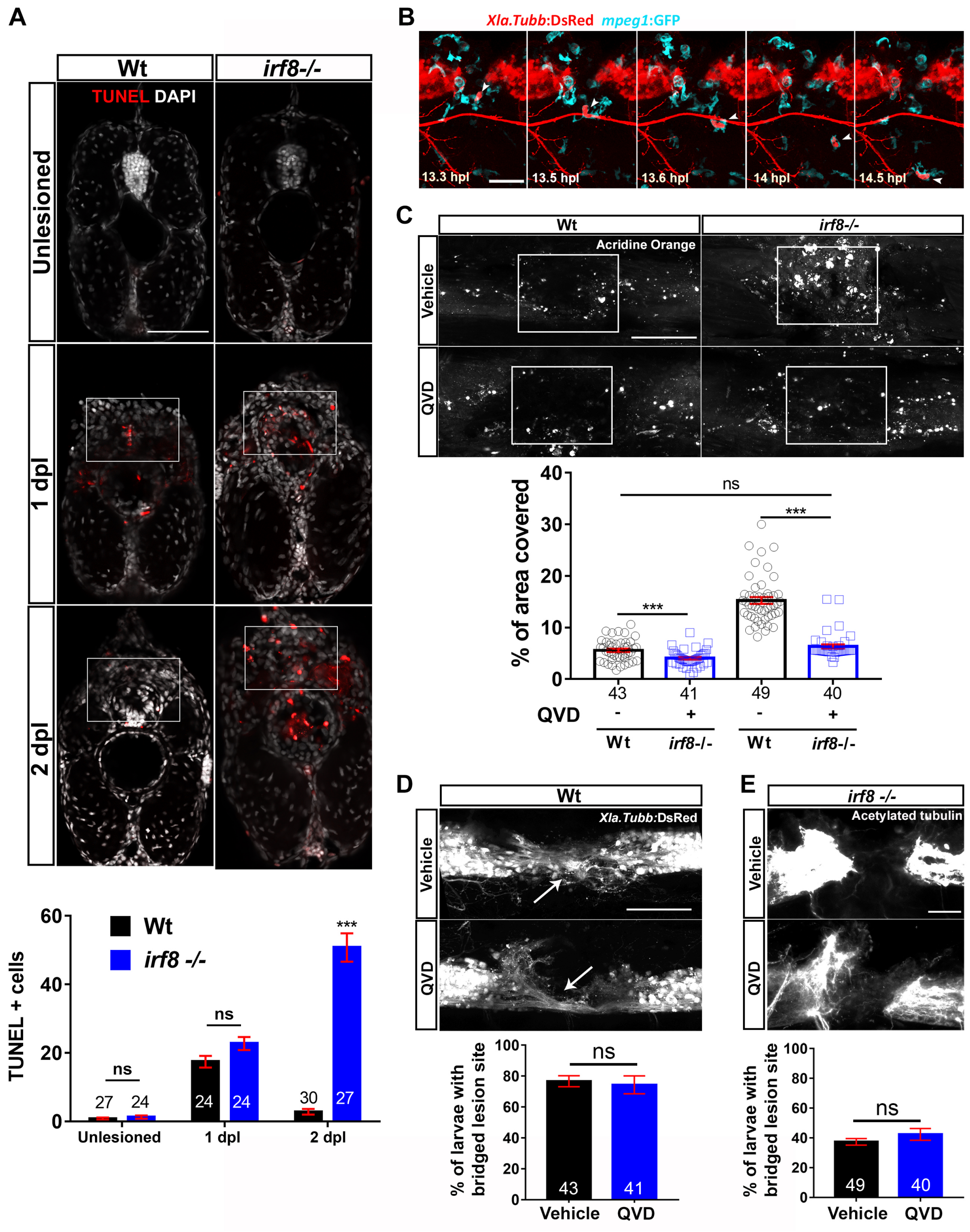

Supplementary Fig. **6:** Debris removal by macrophages is not a major factor for axonal regeneration. **A:** TUNEL labelling on cross sections of larvae (dorsal is up) shows an increased number of TUNEL+ cells in the injury site (indicated by rectangle) of the *irf8* mutant fish at 2 dpl, but not at 1 dpl (*t-test*: ***P<0.001, ns = not significant). **B:** Time-lapse video-microscopy shows that macrophages (cyan, *mpeg1*:EGFP) are in the injury site and remove neuronal debris (*Xla.tubb*:DsRed). Arrowheads point out a macrophage with phagocytosed neuronal material. **C:** Acridine orange labelling at 2 dpl indicates increased levels of debris in the *irf8* mutant, which is reduced to wildtype levels with the pan-caspase inhibitor QVD (*Kruskal-Wallis with Dunn's multiple comparisons post-test*: *** P<0.001, ns indicates no significance). **D,E:** Inhibition of cell death with QVD does not alter regenerative success in wildtype animals (D) or *irf8* mutants (E) at 2 dpl (*Fisher's exact test*: ns indicates no significance). Rectangles indicate regions of quantification and arrows indicate axonal bridges. Lateral views of the injury site are shown (except A); rostral is left. Scale bars: 25µm **A**, 100 µm **B**, **C**, **D, E,** 50 µm. Error bars indicate SEM.

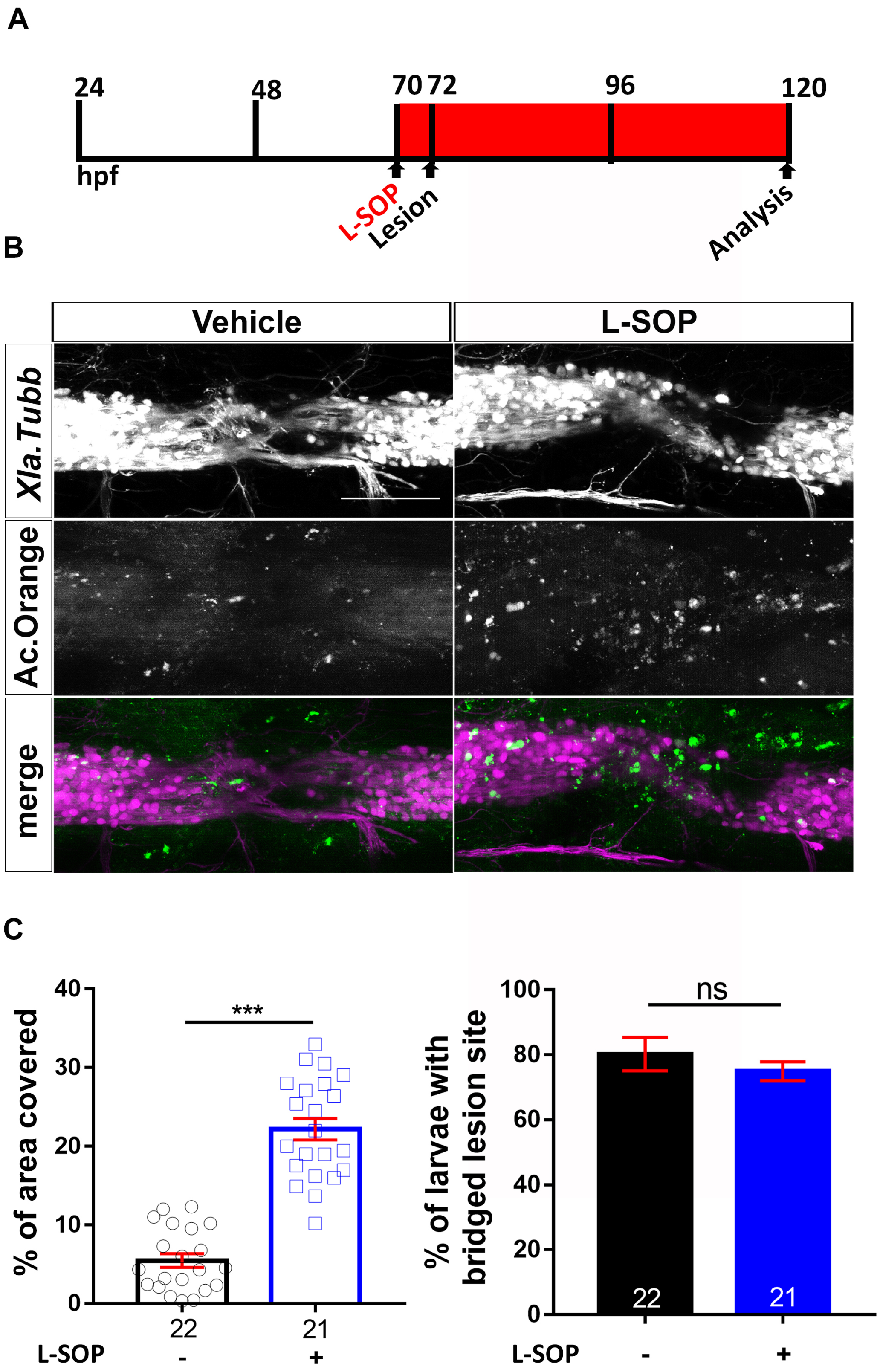

Supplementary Fig. **7:** Treatment with the phagocytosis inhibitor O-Phospho-L-serine (L-SOP) does not affect regenerative success. **A:** The experimental timeline is shown. **B:** Treatment with L-SOP increases the amount of debris, detected with acridine orange (*t-test*: ***P<0.001), but has no effect on axon bridging (*Xla.tubb*:DsRed; *Fisher's exact test*: ns indicates no significance) at 2 dpl. Lateral views of the injury site are shown; rostral is left. Scale bar: 100 µm. Error bars indicate SEM.

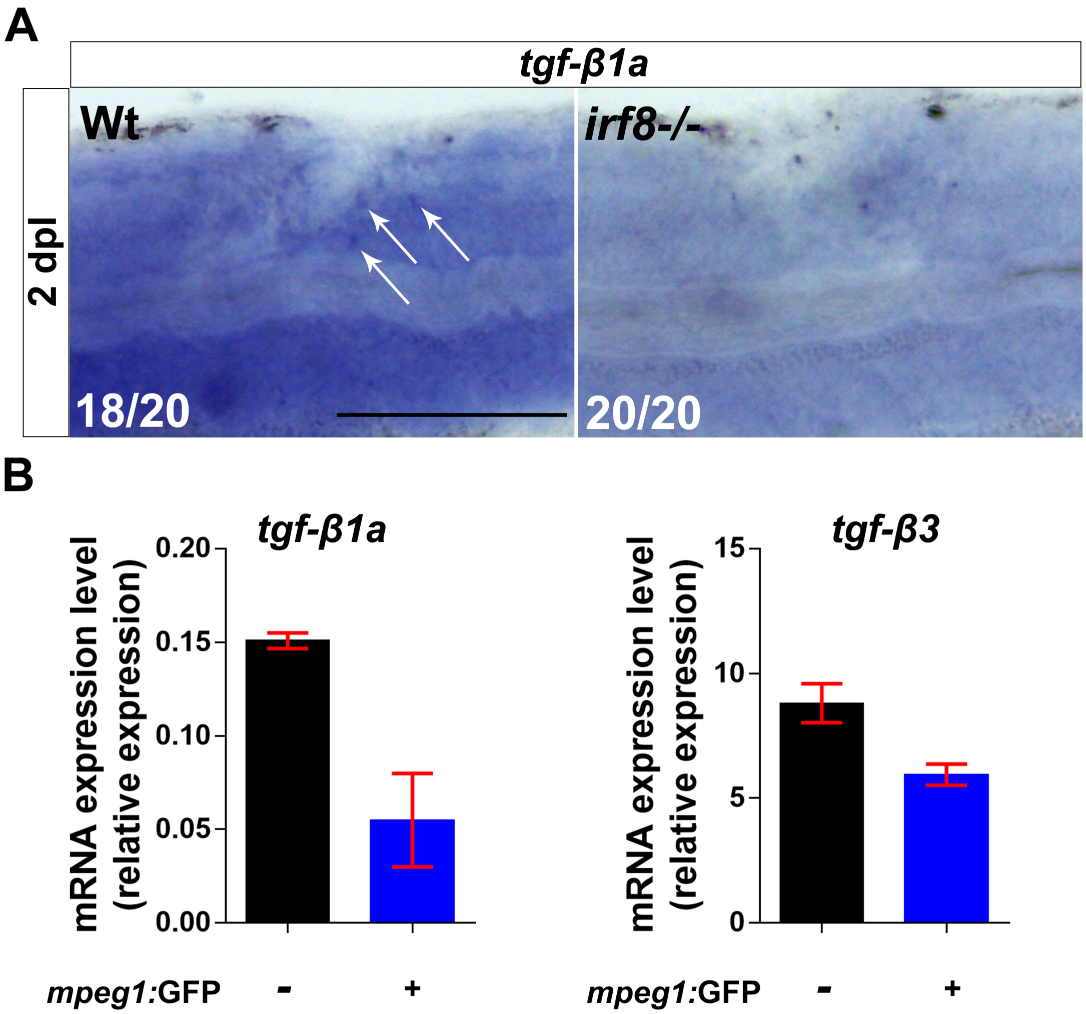

Supplementary Fig. **8:** *tgf-β* is expressed by macrophages and other cell types. **A:** In situ hybridization for *tgf-β1a* shows widespread expression in the lesioned trunk of wildtype and *irf8* mutant larvae at 2 dpl. Arrows show possible macrophages around the injury site of wildtype larvae that are not present around the injury site of *irf8* mutant larvae. **B:** Quantitative RT-PCR using the GFP$^+$ and the GFP$^-$ cell populations of the injury site tissue of lesioned *mpeg1*:GFP larvae shows expression of *tgf-β1a* and *tgf-β3* in purified macrophages (GFP$^+$) and other cell types (GFP$^-$). Lateral views of the injury site are shown; rostral is left. Scale bar: 200 μm. Error bars indicate SEM.

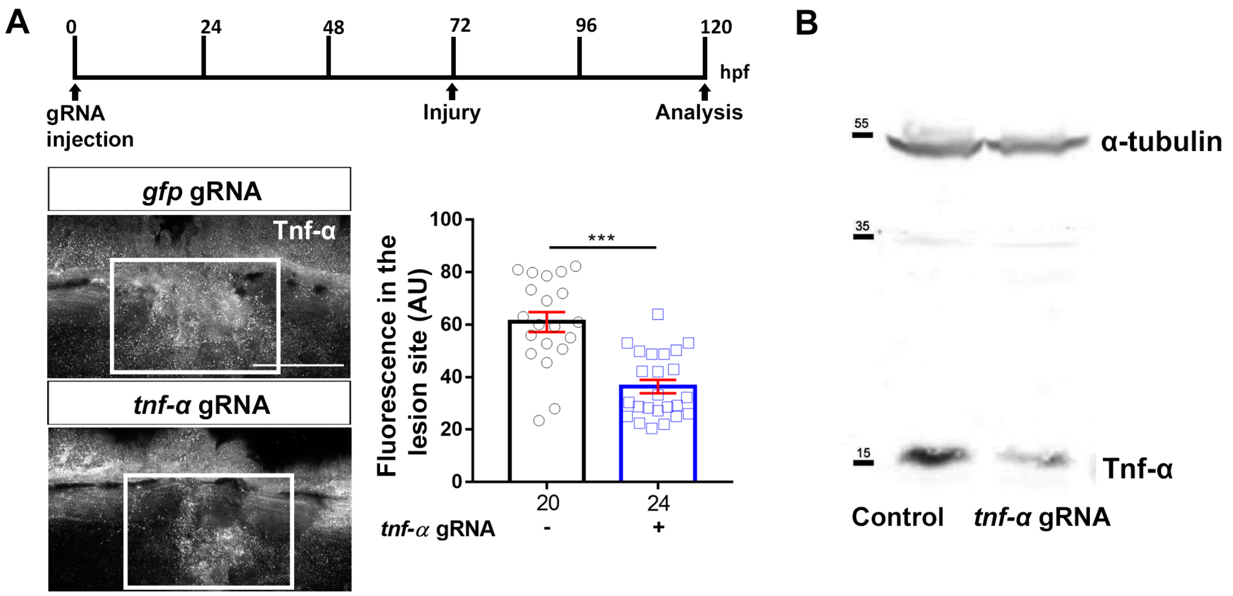

Supplementary Fig. **9:** Tnf-α protein level is reduced after the CRISPR/Cas9 disruption of the *tnf-α* gene. **A:** Immunostaining against Tnf-α shows decreased signal around the injury site (boxed) at 2 dpl (*t-test*: ***P<0.001). AU = arbitrary units. **B:** Western blot against Tnf-α in unlesioned larvae shows decreased protein levels after the gRNA injection. Lateral views of the injury site are shown; rostral is left. Scale bar: 100 µm. Error bars indicate SEM.

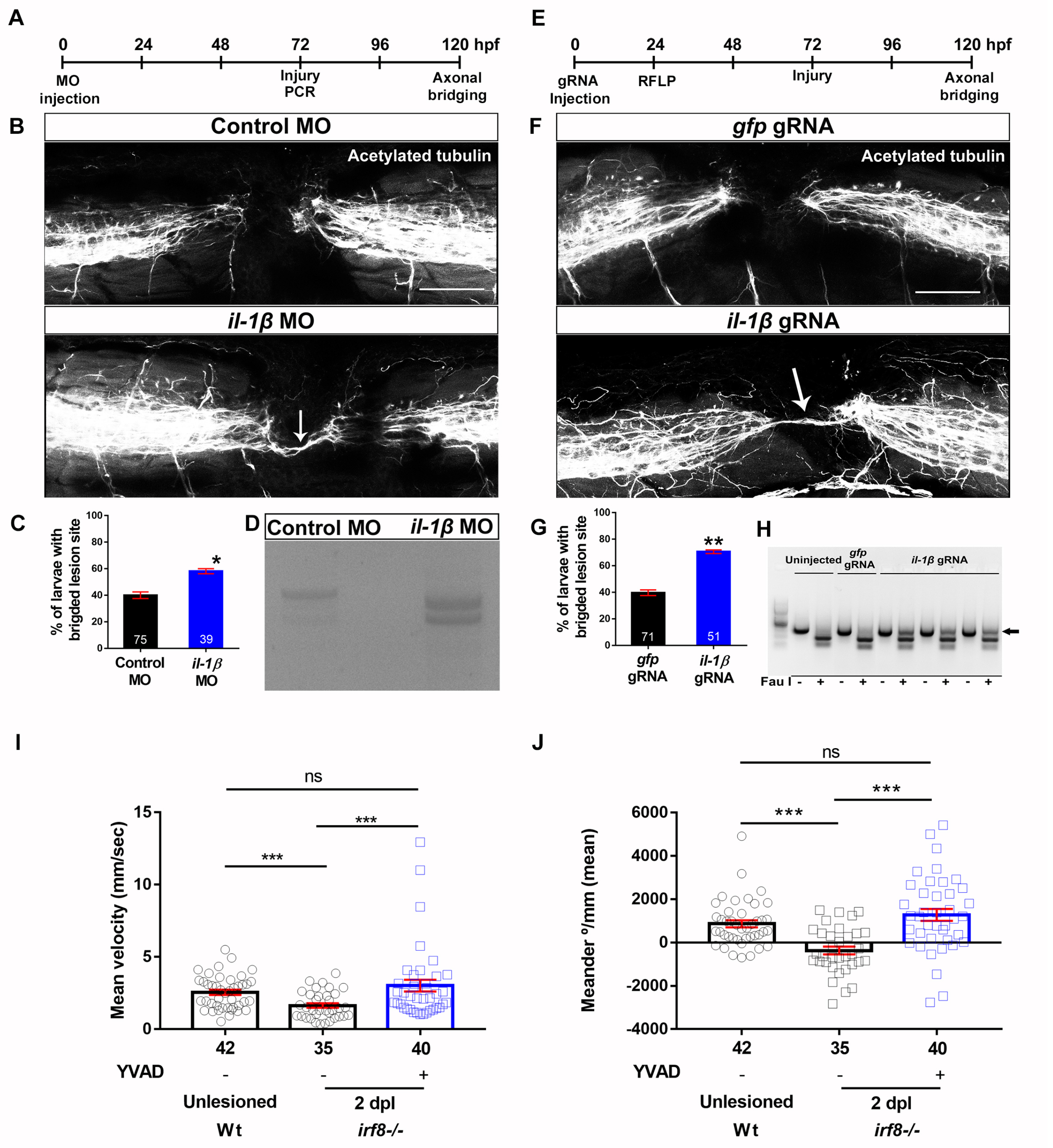

Supplementary Fig. **10:** Inhibition of *il-1β* expression or disruption of the gene rescue axon bridging and parameters of touch-evoked swimming in *irf8* mutants. **A:** The timeline for the morpholino manipulations is given. **B-D:** Morpholino gene knockdown of *il-1β* by injecting into the zygote shows characteristic mis-splicing of *il-1β* mRNA, compared to wildtype animals (D) and partially rescues the bridging phenotype of the *irf8* mutant fish (B,C; *Fisher's exact test*: *P<0.05). **D:** Morpholino gene knockdown of *il-1β* shows characteristic mis-splicing of *il-1β* mRNA, compared to wildtype animals. **E:** The timeline for gRNA manipulation is given. **F-G:** CRISPR/Cas9-mediated disruption of *il-1β* by injecting specific gRNA into the zygote leads to somatic mutations, as shown by RFLP analysis (arrow in **H**) and rescues the bridging phenotype in *irf8* mutants (F,G; *Fisher's exact test*: **P<0.01). **I,J:** YVAD incubation rescues speed (mean velocity) and path shape (meander) that the *irf8* larvae achieve after injury (*Kruskal-Wallis with Dunn's multiple comparisons post-test*: *** P<0.001, ** P<0.01, ns indicates no significance). Lateral views of the injury site are shown; rostral is left. Scale bars: 50 μm. Error bars represent SEM.

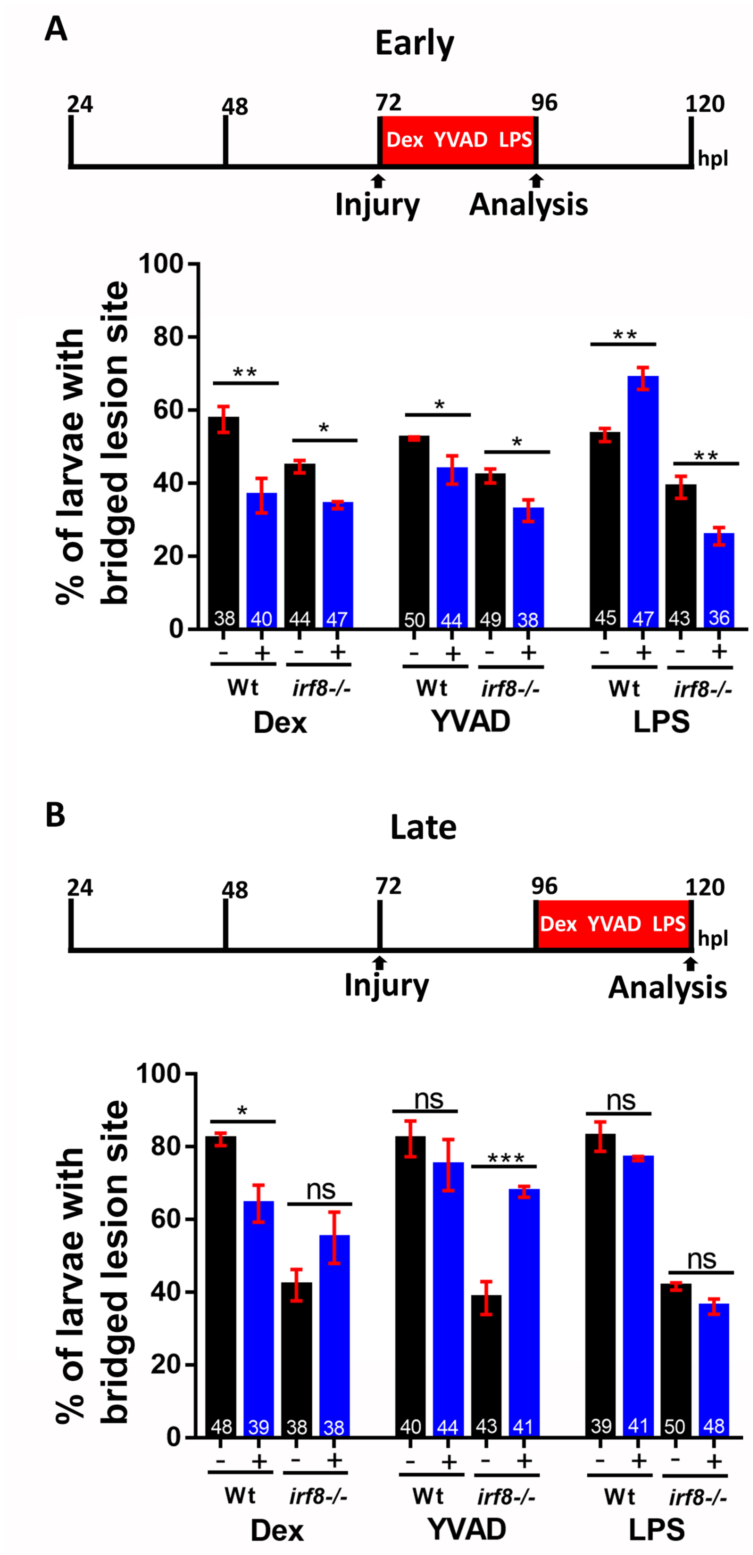

Supplementary Fig. **11:** Temporally restricted manipulations of the immune response reveal time-dependent effects on axon bridging. **A:** During early regeneration, dexamethasone and YVAD treatments impair regeneration in both wildtype animals and *irf8* mutants. Stimulation of the immune response with LPS promotes regeneration in wildtype animals but inhibits it in *irf8* mutants (*Two-way ANOVA followed by Bonferroni multiple comparisons*: $F_{5, 12} = 25.32$. *P<0.05, **P<0.01). **B:** Manipulations during late regeneration show persistent negative effects of dexamethasone in wildtype animals and a strong rescuing effect of YVAD in *irf8* mutants (Two-way ANOVA followed by Bonferroni multiple comparisons: $F_{5, 12} = 16.05$. *P<0.05, ***P<0.001, ns = no significance; Dex = dexamethasone). Error bars represent SEM.

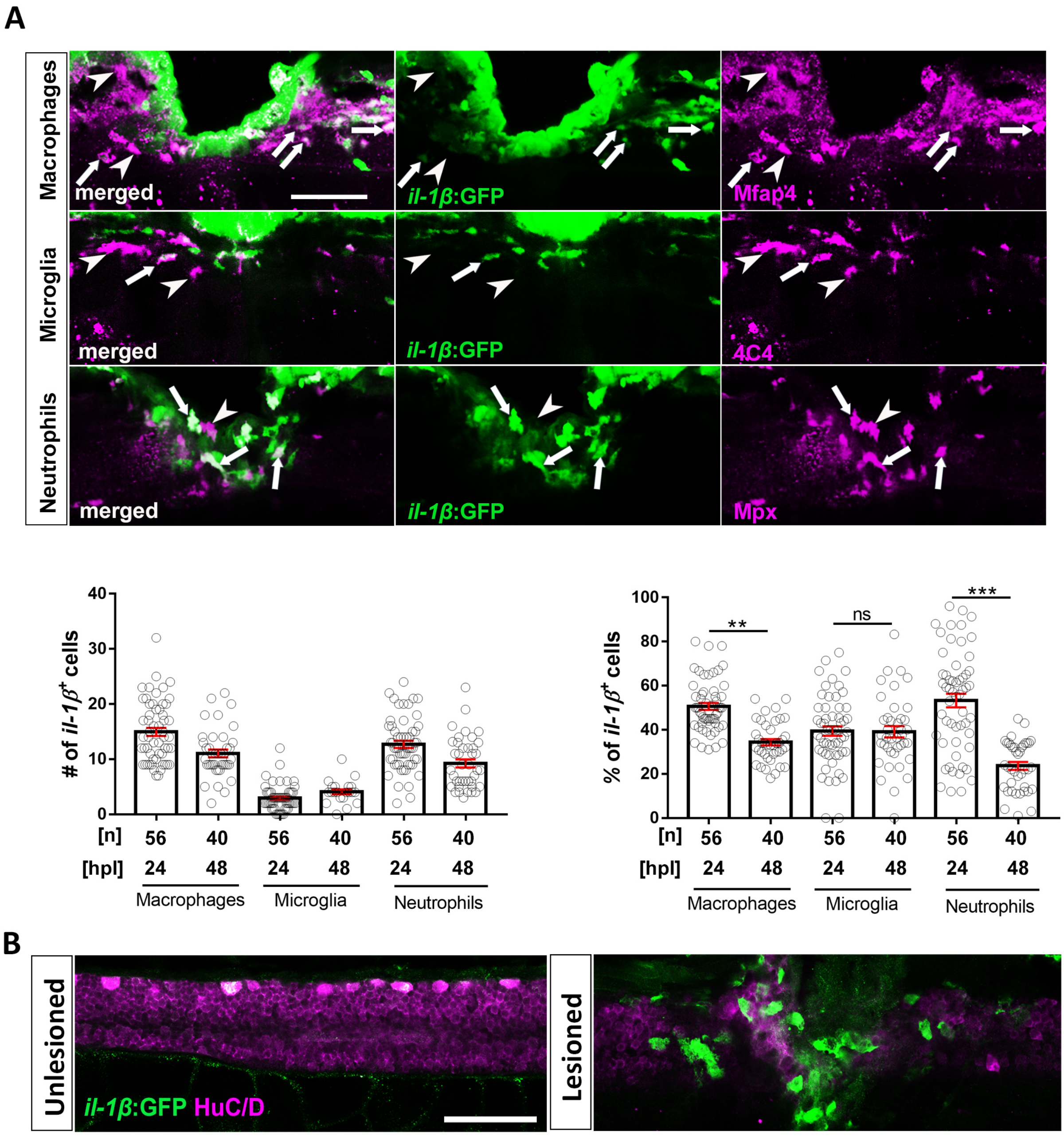

Supplementary Fig. **12:** *il-1β* is expressed in immune cells, but not neurons in wildtype animals. **A:** In the Il-1β reporter line, *il-1β*:GFP^{+} cells are co- labelled (arrows) with the macrophage marker Mfap4, microglial marker 4C4 and the neutrophil marker Mpx. Arrowheads indicate immune cells that are negative for *il-1β*:GFP. The proportion of macrophages (Mfap4^{+}) and neutrophils (Mpx^{+}) that are *il-1β:*GFP^{+} changes over time, whereas the proportion of microglial cells that are *il-1β:*GFP^{+} remains the same (*One-way ANOVA followed by Bonferroni post-test*: $F_{5, 282} = 22.55$, ***P<0.001, **P<0.01, ns = no significance). **B:** *il-1β*:GFP+ cells were rarely co-labelled with the neuronal marker HuC/D at 1 dpl. Lateral views of the injury site are shown; rostral is left. Single optical sections are shown. Scale bars: 50 µm. Error bars represent SEM.

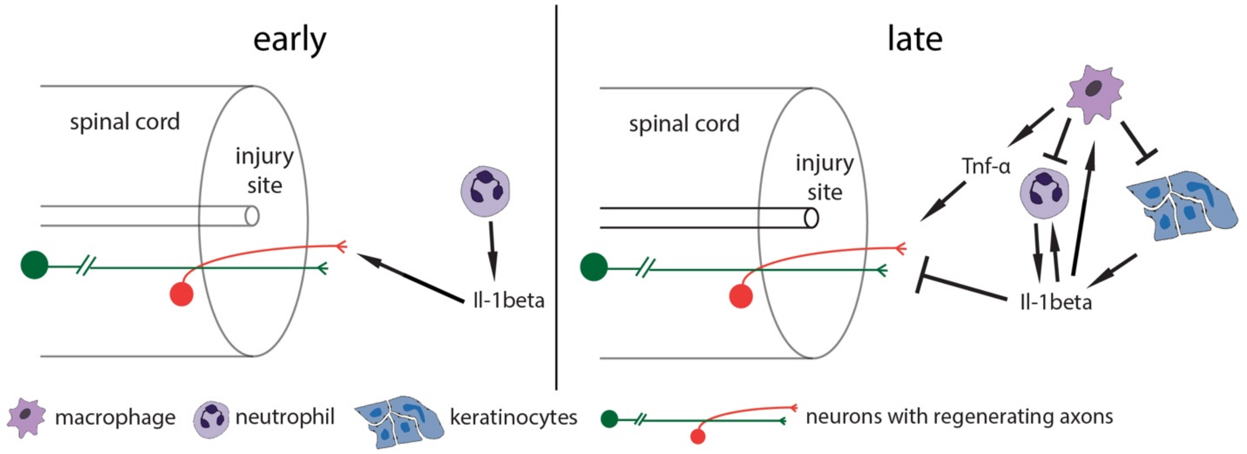

Supplementary Fig.13: Working model of the influence of the innate immune system on axonal regrowth. During the early stage, within hours of inflammation, spinal cord injury triggers neutrophil invasion of the injury site and initiation of the inflammatory response, including *il-1β* production. This initially promotes regeneration but is later strongly inhibitory. At later stages, Il-1β positively regulates presence of neutrophil and macrophages in a feedback mechansims. Macrophages invade the lesion site and down-regulate levels of *il-1β* in neutrophils and basal keratinocytes, while promoting regeneration by releasing Tnf-α. This leads to successful axonal regeneration and recovery of parameters of touch-evoked swimming.
