# Supplementary material for: Dynamic control of proinflammatory cytokines Il-1β and Tnf-α by macrophages in zebrafish spinal cord regeneration
Source: Nat Commun. 2018 Nov 7;9:4670. doi: 10.1038/s41467-018-07036-w (PMC6220182; doi:10.1038/s41467-018-07036-w)
Supplement: Supplementary file 5 — Description of Additional Supplementary Files [file 41467_2018_7036_MOESM5_ESM.docx]

**Title:** Supplementary Movie 1
**Description:** mpeg1:mcherry/mpx:gfp double transgenic animals, showing the recruitment of macrophages and neutrophils to the injury site. Timelapse imaging was performed for 5 h starting at 24 hpl with 10 minutes interval between images.

**Title:** Supplementary Movie 2
**Description:** Xla.Tubb:DsRed/mpeg1:EGFP double transgenic animals, showing macrophage phagocytosis of neuronal debris and axonal bridging with no interaction between immune cells and axons. Time-lapse imaging was performed for 16 h starting at 6 hpl with 10 minutes interval between images.
